# Changes in protein abundance are observed in bacterial isolates from a natural host

**DOI:** 10.3389/fcimb.2015.00071

**Published:** 2015-10-14

**Authors:** Megan A. Rees, Timothy P. Stinear, Robert J. A. Goode, Ross L. Coppel, Alexander I. Smith, Oded Kleifeld

**Affiliations:** ^1^Coppel Laboratory, Department of Microbiology, Monash UniversityClayton, VIC, Australia; ^2^Monash Biomedical Proteomics Facility, Department of Biochemistry and Molecular Biology, Monash UniversityClayton, VIC, Australia; ^3^Stinear Laboratory, Department of Microbiology and Immunology, University of MelbourneParkville, VIC, Australia

**Keywords:** natural infection, *Corynebacterium pseudotuberculosis*, dimethylation labeling, bacterial response to host, lymph nodes, thiopeptide, carbon starvation protein A, lactate utilization

## Abstract

Bacterial proteomic studies frequently use strains cultured in synthetic liquid media over many generations. It is uncertain whether bacterial proteins expressed under these conditions will be the same as the repertoire found in natural environments, or when bacteria are infecting a host organism. Thus, genomic and proteomic characterization of bacteria derived from the host environment in comparison to reference strains grown in the lab, should aid understanding of pathogenesis. Isolates of *Corynebacterium pseudotuberculosis* were obtained from the lymph nodes of three naturally infected sheep and compared to a laboratory reference strain using bottom-up proteomics, after whole genome sequencing of each of the field isolates. These comparisons were performed following growth in liquid media that allowed us to reach the required protein amount for proteomic analysis. Over 1350 proteins were identified in the isolated strains, from which unique proteome features were revealed. Several of the identified proteins demonstrated a significant abundance difference in the field isolates compared to the reference strain even though there were no obvious differences in the DNA sequence of the corresponding gene or in nearby non-coding DNA. Higher abundance in the field isolates was observed for proteins related to hypoxia and nutrient deficiency responses as well as to thiopeptide biosynthesis.

## Introduction

*Corynebacterium pseudotuberculosis* caseous lymphadenopathy and wasting in sheep and goats (Dorella et al., 2006) resulting in significant mortality and morbidity as well as economic costs to livestock (Bush et al., 2012). Human cases of *C. pseudotuberculosis* infection are rare but well documented (Peel et al., 1997; Trost et al., 2010) and have similar clinical features and pathology.

After inoculation through a skin wound *C. pseudotuberculosis* establishes a chronic caseating infection in the lymph nodes of its animal hosts (Baird and Fontaine, 2007; Fontaine and Baird, 2008). This space is rich in immune cells and necrotic material, which contains degradative enzymes and cellular waste products. *C. pseudotuberculosis* and related species are known to utilize both carbohydrate and lipid carbon sources for growth (Inui et al., 2004), and have the capacity to switch between these metabolic pathways depending on substrate availability (Woo et al., 2010). Bacterial cells exposed to this hostile environment may counter this by modifying the proteins expressed on their surface (Rees et al., 2015). It is likely that longer term exposure to such conditions will modify the expression of different groups of proteins throughout the whole bacterial proteome, and proteome differences have been noted in direct comparisons between bacteria isolated from the host and media (Weigoldt et al., 2011). However, it is not clear whether this would occur from changes in the genome or the regulation of protein transcription (Güell et al., 2011).

The repertoire of proteins expressed by an organism will differ depending on the cell's growth phase and surrounding environment. The host environment presents specific challenges to a pathogen that includes both targeted attack from the host immune system and general stressors which arise from the physical milieu of the host cells in which the bacteria reside such as hypoxia, acidosis and paucity of nutrients.

We set out to determine if the protein repertoire of bacteria recently isolated from the host after a sustained infection differed from that of cells that had been passaged through a liquid media environment. To do this we compared their genomes and utilized quantitative proteomics to compare abundance of individual protein species when growing in common culture media.

## Materials and methods

### Collection of bacterial isolates

Three field isolates of *C. pseudotuberculosis* were obtained and are detailed in Table 1. Specimens of macroscopically infected lymph nodes from three separate randomly selected sheep were obtained from a local abattoir (Herd Abattoir, Geelong). Infected caseous material from the lymph nodes were streaked onto BHI agar plates and an individual colony of bacteria was selected for subculture and sequencing. *C. pseudotuberculosis* was grown in BHI media aerobically at 37°C, with continuous shaking. Growth of bacteria in liquid media was measured by determining cell mass with optical densitometry (OD 400 nanometers).

**Table 1:** **Summary of isolates investigated with details of whole genome sequencing approaches used and results, including sequencing approach, and genome assembly**.

**Sequencing statistics**	**Bacterial isolate sequenced**			
	**Cptb_RLC_001**	**Cptb_RLC_002**	**Cptb_RLC003**	**Cptb_C231**
Sequencing instrument	Ion torrent	MiSeq	MiSeq	MiSeq
Number of contig		42	27	57
Total contig length (bp)		2231996	2323242	2317599
Minimum contig length (bp)		221	321	287
Average contig length (bp)		53142	86046	40659
Maximum Contig Length (bp)		333978	385019	152746
N50 (bp)		205882	235822	78603
Reads	3507459		
Mean length (bp)	183		
Assembly type	Scaffold	*De novo*	*De novo*	*De novo*

*Contig, Continous set of overlapping DNA; bp, base pairs*.

A laboratory reference strain of *C. pseudotuberculosis* C231 (Cptb_C231; Burrell, 1978; Ruiz et al., 2011) was obtained from Dr Rob Moore, Commonwealth Scientific and Industrial Research Organisation (CSIRO), Geelong, Australia. This strain was isolated more than 30 years ago and had been only passaged in liquid BHI over this time.

### Whole genome sequencing

Genomic DNA extraction was performed with the Nucleon Kit (Amersham Biosciences), and this method generally followed the manufacturer's instructions. Specifically, *C. pseudotuberculosis* grown on BHI Agar plates without antibiotics and were disrupted with acid washed glass beads by bead beater (Fastprep, MP Biomedicals), in the presence of RNase and Proteinase K. Genomic DNA was separated from proteins by suspension in sodium percholate and chloroform prior to precipitation in Nucleon resin. Genomic DNA was secondarily precipitated with 100% ethanol prior to final re-suspension in water.

For the first field isolate Cptb_RLC001 high throughput sequencing was performed on this genomic material using the Ion Torrent Personal Genome Machine (Life Technologies, Guilford, CT, USA) with a 316 chip and 200 bp sequencing chemistry. The sequence reads were mapped to the *C. pseudotuberculosis* C231 reference genome using SHRiMP 2.2 (Rumble et al., 2009). SNPs were identified using Nesoni v0.70, to construct a tally of putative differences at each position that included substitution mutations only (www.vicbioinformatics.com).

Genome sequences for isolates (Cptb_RLC002 and RLC003) were obtained using an Illumina MiSeq with Nextera XP library preparation and 2 × 300 bp sequencing chemistry to approximately 200x read coverage. We also sequenced our version of the *C. pseudotuberculosis* C231 reference (GenBank reference NC_017301). Resulting DNA sequence reads and existing sequence reads for Cptb_RLC001 were analyzed as previously described (Rees et al., 2015) to define a core genome by aligning reads to the 2,328,208 bp C321 reference chromosome. A genome for each isolate sequenced using Illumina chemistry was partially assembled *de novo* using Velvet v1.20.10 (PMID:18349386), with the resulting contigs annotated with Prokka v1.10 (PMID:24642063). The accessory genome for each of the isolates was explored using Fripan (http://drpowell.github.io/FriPan/) with ortholog clustering inputs obtained from Proteinortho5 (PMID: 21526987) with the following match parameters, expect score = 1e-09, identity = 80%, coverage = 30%. The translated protein coding DNA sequences predicted by Prokka were used as inputs to Proteinortho5.

### Whole proteome extraction

Cultures of *C. pseudotuberculosis* (both reference strain Cptb_C231 and three field isolates Cptb_RLC001, Cptb_RLC002, Cptb_RLC003) were grown in BHI liquid media with shaking until late exponential phase (*OD* = 15) then washed three times with PBS and pelleted, resulting in a final volume of packed cells of 100 μl, these were then frozen at −80°C until required.

Whole Proteome Extracts were Prepared by Two Methods.

Firstly, unlabeled samples were prepared for label free quantitative analysis using the FASP method adapted from Wisniewski (Wiśniewski et al., 2009) with some modifications. Specifically 100 μl of washed and packed bacterial cells were freeze thawed then suspended in three times volume lysis buffer (4% SDS with 100 mM Tris plus 100 mM DTT) and heated to 95°C for 5 min. Samples were sonicated for 5 min and cellular debris was precipitated. Proteins were quantified by Bradford assay (Bradform Ultra, Expedion). Then 100 μg of bacterial lysate were combined with 8 M Urea in 100 mM Tris-HCl, (total volume 200 μL) loaded on 30 kD ultrafiltration device (Millipore) and centrifuged at 14,000xg for 15 min, a further 200 μL of 8 M Urea in 100 mM Tris-HCl was added and centrifuged at 14,000xg and this was repeated. Then 100 μL of 0.05 M iodoacetamide in 8 M Urea in 100 mM Tris-HCl was added and incubated for 20 min prior to centrifugation and washing with 8 M Urea in 100 mM Tris-HCl. The sample then underwent overnight digestion with trypsin and the digests were collected in 75 μL of 50 mM ammonium bicarbonate by centrifugation and the filter device rinsed with 50 μL 0.5 M NaCl and centrifuged. The filtrate now contains the digested peptides and this solution was acidified to pH 2 with formic acid.

Desalting and concentration of peptides in solution was performed with bench-top columns [TopTip™ Reversed Phase (C-18), Glygen] prior to loading for HPLC-MSMS analysis. The columns were used according to manufacturer's instructions; specifically solvents used included a binding solution of 0.1% formic acid in MilliQ water and a releasing solution of 0.1% formic acid in 60% acetonitrile. A 100 μL aliquot of sample was then added to the column with flow through sample recaptured. The column was then washed three times with 50 μL aliquot of binding solution then peptides for analysis were released with two 50 μL aliquots of releasing solution. The solvent was then evaporated and peptides resuspended in 20 μL 2% acetonitrile with 0.1% formic acid and sonicated prior to mass spectrometry analysis.

The second method involved labeling samples with dimethylation for quantitative analysis. Bacterial samples were brought to room temperature and added to 400 μl of a lysis buffer consisting of 1% Sodium Deoxycholate (SDC; Sigma) 10 mM Tris (2-carboxyethyl) phosphine hydrochloride (TCEP; Sigma), 40 mM 2Chloroacetomide (CAA; Sigma Aldrich) in 100 mM HEPES at pH 8.1. Cells were vortexed in lysis buffer then were heated to 95°C for 5 min.

Cells were disrupted with 10 um amplitude probe sonication for three rounds of 30 s, with cooling between cycles. Samples were diluted with 500 μL milliQ water to a final volume of 1 ml. Protein concentration was then determined with BCA kit (Pierce™ BCA Protein Assay Kit, Thermo Scientific). Trypsin (Promega) was added to a final enzyme-to-protein ratio of 1:100 and the samples were digested overnight at 37°C.

Samples were then labeled by dimethylation with the reference strain C231 labeled with light (^12^CH_2_) formaldehyde and the field isolates labeled with heavy (^13^CD_2_) formaldehyde (Cambridge Isotope Laboratories). Prior to labeling the pH of each sample was adjusted to seven using formic acid, then either 2 M of light or heavy formaldehyde was added to the respective samples to reach final concentration of 40 mM followed by addition of 1 M NaBH_3_CN to final concentration of 20 mM. Samples were vortexed and left shaking at room temperature overnight to complete the labeling process. The reaction was quenched with the addition of 1 M glycine to each tube (final 100 mM). An aliquot of each sample (500 μL) was diluted further with 1.5 ml milliQ water, each field isolate was mixed with the same volume of reference strain, following which samples were acidified with formic acid to pH ~2.8. SDC was removed using phase transfer (Masuda et al., 2008). The resulting peptide mixture was fractionated using strong cation exchange cartridge (Bond Elut Plexa PCX, Aglient) with serial elutions of solutions of increasing concentrations of ammonium acetate. The strong cation exchange cartridge was activated with 1 ml of methanol then washed with 1 mL of washing buffer containing 50% (w/v) ethyl acetate, 0.5% (v/v) formic acid, and 50% milliQ water. Samples containing peptides were then loaded onto the cartridge and washed with the same washing buffer three times. Cartridges were then washed with a solution of 0.1% (v/v) formic acid three times. Then samples were eluted sequentially with solutions containing 20% acetronitrile (v/v) and 0.5% formic acid and a variable amount of ammonium acetate (100, 150, 200, 250, 300 mM). The final elution was performed with 80% (v/v) acetonitrile and 5% (v/v) ammonium hydroxide. The solvent was then evaporated with a centrifugal evaporator and then peptides were resuspended in 20 μL 2% acetonitrile with 0.1% formic acid and sonicated in a water bath sonicator for 10–15 min prior to mass spectrometry analysis.

### Mass spectrometry

Samples were analyzed by LC-MS/MS using a Q Exactive™ Orbitrap mass spectrometer (Thermo Scientific) coupled online with a RSLC nano-HPLC (Ultimate 3000, Thermo Scientific) to derive mass spectra of individual peptides and peptide fragment ions were identified with tandem mass spectrometry (MS/MS). Samples were injected onto a Thermo RSLC pepmap100, 75 um id, 100 Å pore size, 50 cm reversed phase nano column with 95% buffer A (0.1% formic acid) at a flow rate of 300 nL/min. The peptides were eluted over a 60-min gradient to 40% buffer B (80% Acetonitrile 0.1% formic acid). The eluate was nebulised and ionized using the Thermo nano electrospray source coated silica emitter with a capillary voltage of 1700 V. Peptides were selected for MS/MS analysis using Xcalibur software (Thermo Finnigan) in Full MS/dd-MS^2^ (TopN) mode with the following parameter settings: TopN 10, MSMS AGC target 5e4, 120 ms Max IT, NCE 27 and 2 m/z isolation window. Dynamic exclusion was set to 30 s. Generally a single injection was performed for each experimental preparation replicate and two blank injections between each experimental sample.

### Analysis of mass spectral data

The quantitative studies described below were included comparison of three biological repeats of the reference strain Cptb_C231 to each of the field isolates. MS label-free and dimethylation data were analyzed with the MaxQuant software (Cox and Mann, 2008) Version 1.5.2.8. Search parameters included specific digestion with Trypsin with up to two missed cleavages: Protein N terminal acetylation (protein) and methionine oxidation were set as variable modifications while cysteine alkylation was set as fixed modfication. The searches were performed against a combined database (Supplementary File, CombinedProteomeDatabase_CptbMerge) generated from all sequenced and annotated genomes of *C. pseudotuberculosis* Cptb_C231 and the field isolates *C. pseudotuberculosis* Cptb_RLC001, RLC002 and RLC003, and Cptb C231 sequences downloaded from UniProt (March 2015 version). For label-free analysis the MaxQuant search include the “LFQ” option. Search and quantification using dimethylation were using two labels option using light and heavy dimethylation modification on peptide N-termini and lysine residues. Statistics and further analysis were performed with Perseus framework (version 1.5.1.6). Significant label-free changes were determined using two-sample *T*-test, while dimethylation significant changes were determined using “Significant A” test (Cox and Mann, 2008).

Likely protein functions were assigned by the COG database (Tatusov et al., 2001), proteins sequence data was searched against the COG target HMM database/bio/db/hmmer3/COG.hmm a database of hidden Markov Models using the hmmpfam program from the HMMER software suite (http://selab.janelia.org/software.html) with predicted orthologous functional group from COG and NCBI. The Inparanoid program (Remm et al., 2001; O'Brien et al., 2005), which is based on reciprocal BLAST, was used for prediction of homology with *M. tuberculosis*.

## Results

**Figure 1:**
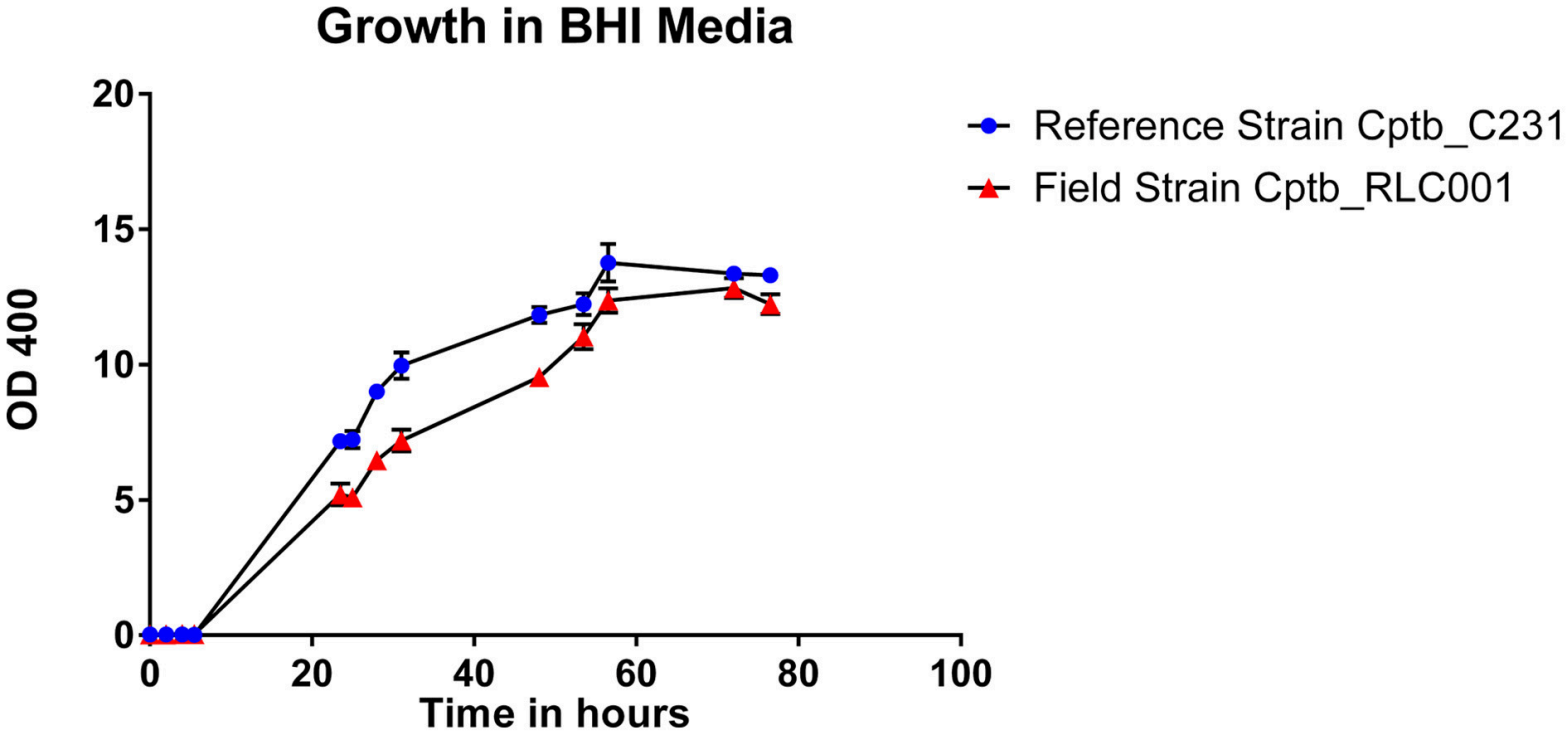
**Graph demonstrating growth rates in BHI liquid media of a reference strain of *C. pseudotuberculosis* Cptb_C231 (blue circles) compared to that of the field isolate *C. pseudotuberculosis* Cptb_RLC001 (red triangles)**. The chart plots relative cell density in liquid media, as measured by optical density (OD 400), against time in hours. Mean values plotted with error bars representing standard error of the mean. This is a representative growth curve with identical features seen in other field strains.

**Figure 2:**
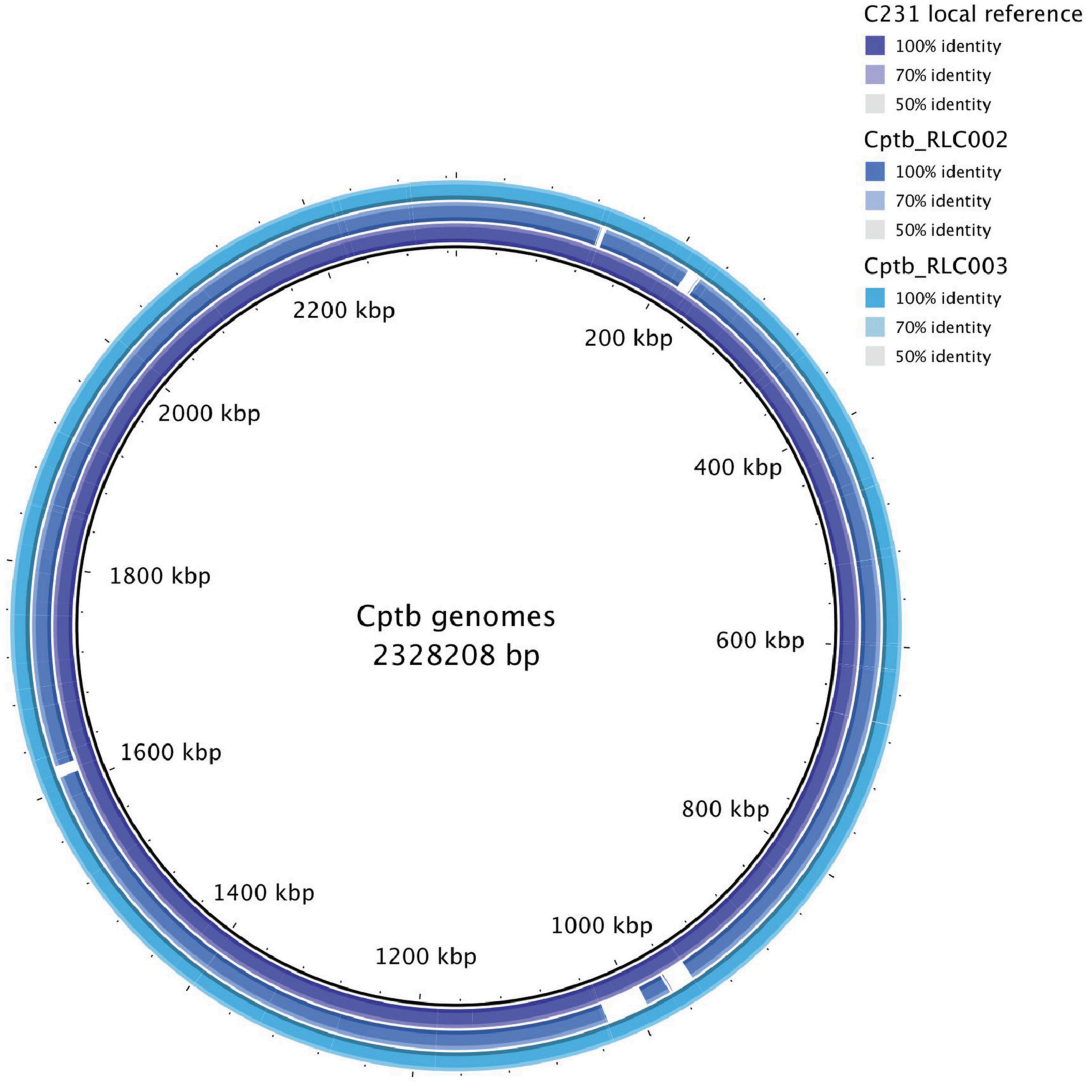
**Map of whole genome sequencing results demonstrating similarity or identity between the reference strain Cptb_C231 and the field isolates Cptb_RLC002 and Cptb_RLC003**.

The growth of bacteria in liquid BHI media as measured by optical density was slightly reduced in the field isolate Cptb_RLC001 compared to the reference strain Cptb_C231; this is shown in Figure 1. A summary of genome sequencing results for each strain is listed in Table 1. The whole genome of this isolate was determined (Rees et al., 2015), and comparison of Cptb_RLC001 to the reference genome of *C. pseudotuberculosis* Cptb_C231 identified a total of 62 single-nucleotide polymorphisms (SNPs), of which 44 were in coding regions of 38 proteins. Complete genome sequencing of the reference strain Cptb_C231 and the field isolates Cptb_RLC002 and Cptb_RLC_003 demonstrated a high level of similarity between each of the isolates with very few coding sequences absent from the field isolates. This is shown in Figure 2. The vast majority of genes sequenced were found in all four isolates with 1878 genes encoding predicted proteins present in all four isolates (88% of all genes identified), 227 genes present in three of the four isolates and a further 19 genes present in only two isolates.

**Figure 3:**
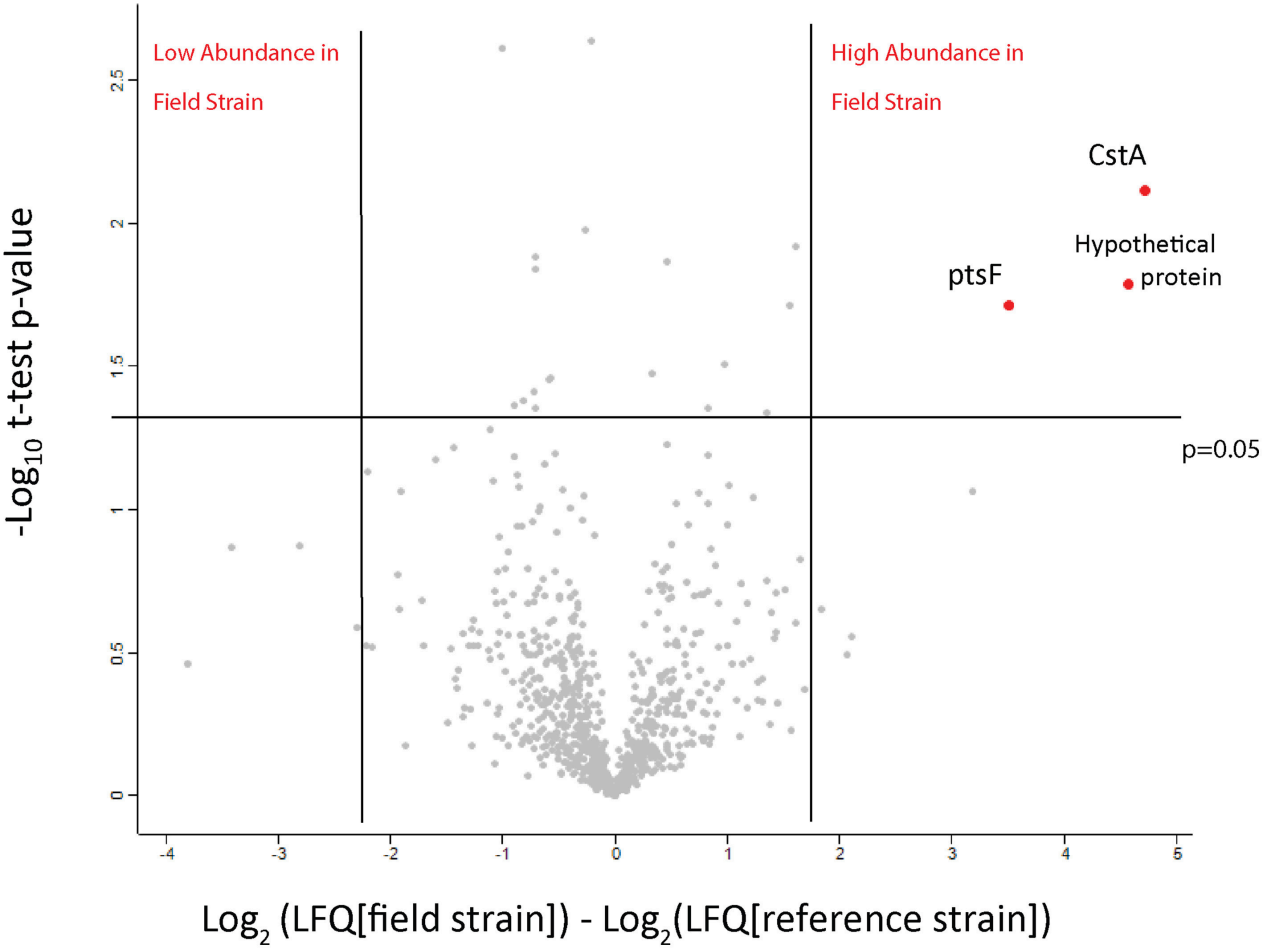
**Volcano plot of the observed protein abundance changes by label-free quantification**. The protein expression ratio of protein in the field isolates to the reference strains in label-free quantification were plotted against the −log_10_ of the probability calculated by *t*-test. Outliers of *p* = 0.05 and expression fold different (in log_2_ scale) marked by blue lines.

The paucity of substantial genomic variation and the different growth rates of the field strains prompted a comparison of these isolates' proteomes. Initially, we tested the possibility of using quantitative proteomics to detect differences in protein abundance between the isolates. We performed a small scale comparison based on label-free quantification in order to compare the two out of the three field isolates to Cptb_C231 after growth in BHI media. All together over 1250 protein groups were identified and for about 830 of them we were able to obtain a quantitative comparison (Figure 3, and details in Supplementary Table 1, ReesLFQalldb.xlsx) by utilizing label-free quantification based on comparison of peptide numbers and intensity (Cox et al., 2014). Three proteins were found to be significantly more abundant in the field isolates in comparison to the Cptb_C231 (Figure 3, marked in red). These proteins included carbon starvation protein A (pcsA), and PTS system fructose-specific EIIABC component (pstF) and a 42 amino acid long uncharacterized protein GI:503006038 (marked as Cptb_C231_00414, Cptb_RLC_001 _02057, Cptb_RLC_002_01413, and Cptb_RLC_003_00551 in the sequencing data–Supplementary Table 3, CombinedProteomeDatabase_CptbMerge). Blast search of this sequence against NCBI NR database reveal that the latter protein is unique to several strains of *C. pseudotuberculosis* and *C. ulcerans* (data not shown). As indicated by the MS/MS search results GI:503006038 is not listed in UniProt database for Cptb_C231 but this gene appeared in all the four sequences we obtained.

Encouraged by these results we set out to perform a more comprehensive comparison of Cptb_C231 and the field isolate. We utilized dimethylation labeling which allows for simultaneous MS analysis and direct and accurate quantification of the compared proteomes. A total of 1358 *C. pseudotuberculosis* proteins were identified in these samples, of which it was possible to reliably determine the ratio between Cptb_C231 and at least one field isolate for 1354 proteins (Supplementary Table 2, ReesDiMet.xlsx). This represents good proteome coverage, with 65% of the 2091 proteins predicted from the encoding genome detected. Transcriptional profiling of *M. tuberculosis* during chronic infection in a mouse model found that 50% of the genome was actively transcribed (Talaat et al., 2007). Therefore, the proteome profiling and quantitative information obtained here most probably capture most of the proteins expressed by the organism at a specific point in time.

**Figure 4:**
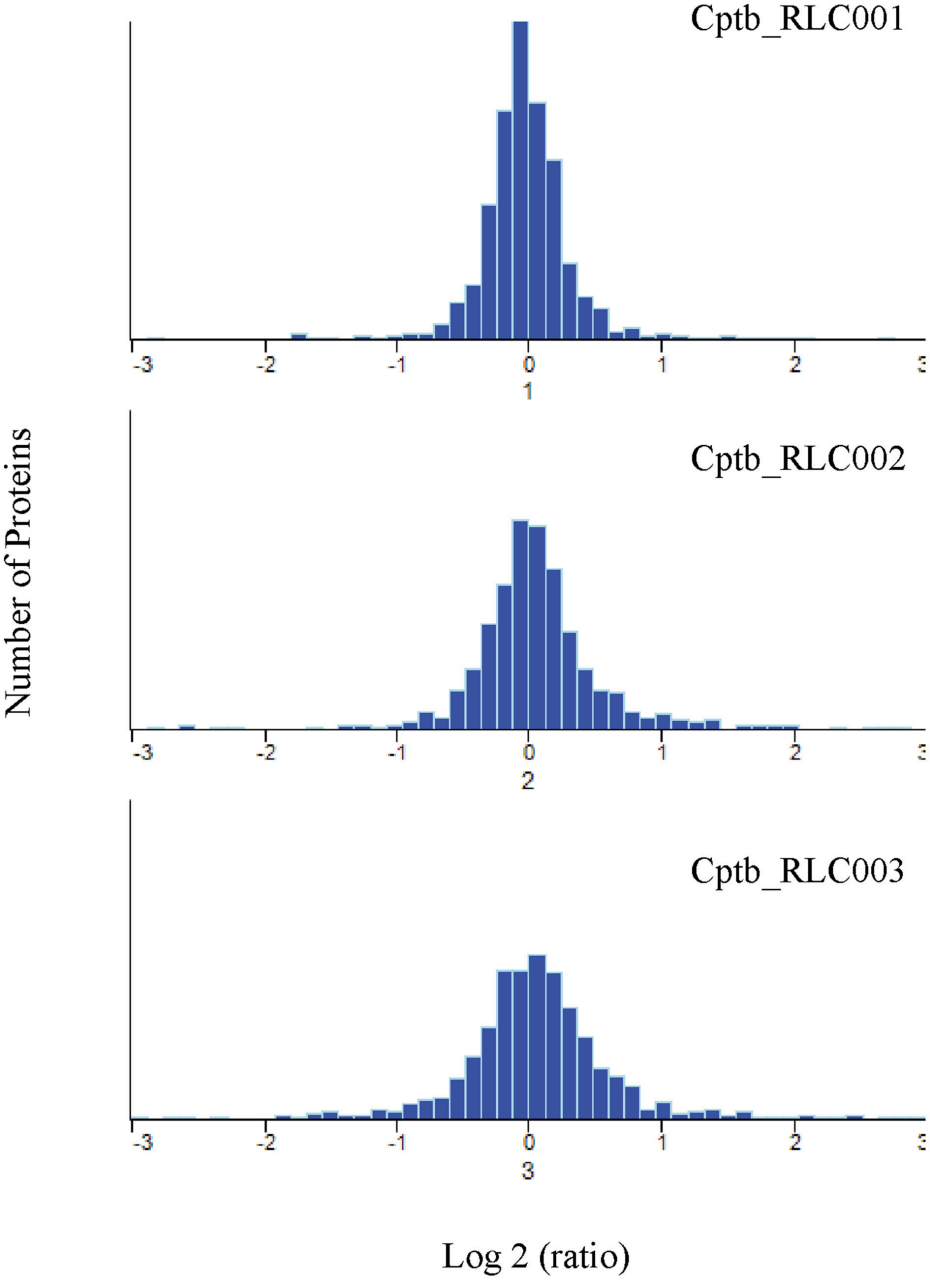
**Quantitative comparison by dimethylation**. Frequency distribution of Log2 heavy/light ratio for the identified protein groups, indicating similar but not identical protein expression levels for field isolates and reference strain.

**Figure 5:**
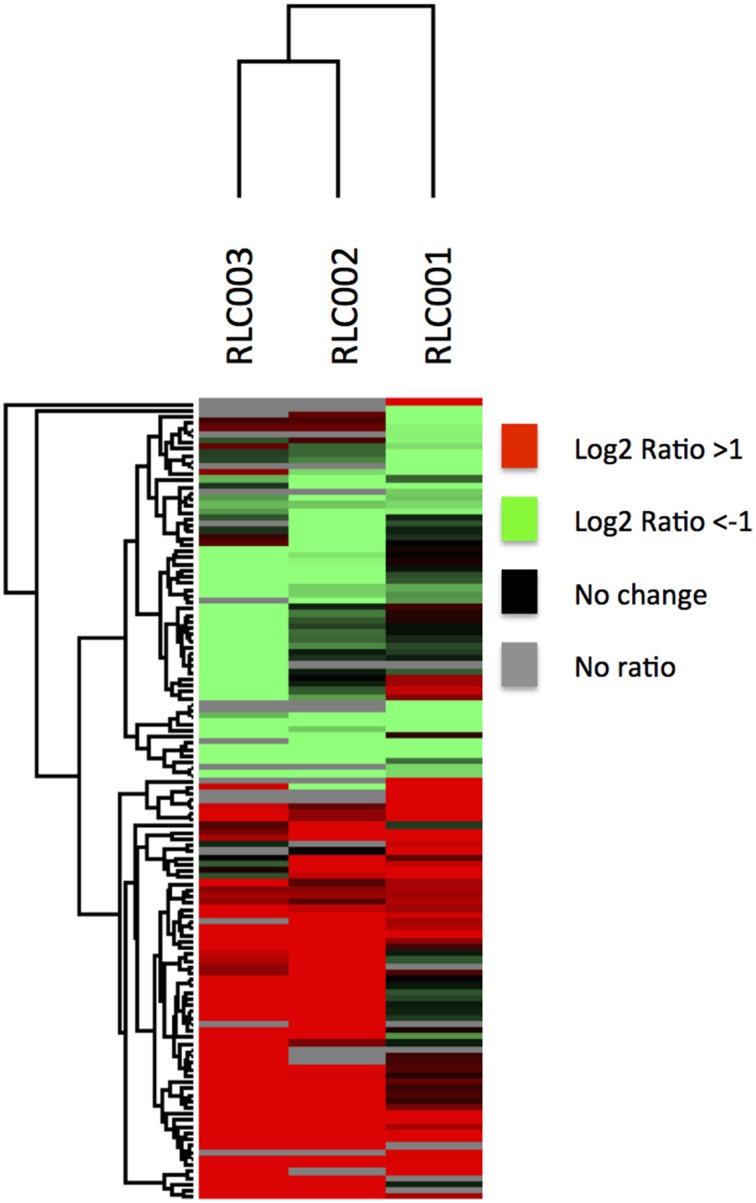
**Quantitative comparison by dimethylation**. Hierarchical clustering of proteins with significantly altered expression in field isolates relative to the reference strain. The map is color coded to show proteins of increased abundance in the field strain in red, decreased abundance in green, not changed in black and not identified in gray. Some proteins shared very similar expression profiles across all field isolates despite their different origins.

When comparing the relative expression levels of proteins in the field isolates to the reference strain, the vast majority of proteins were at similar abundance spanning from log 2 of −0.5 to 0.5 (Figure 3). The overall distributions of the protein ratios for the three different strains are similar but not identical, which might reflect that these isolates originate from infections of different animals. A similar distribution of protein abundance was seen between each of the three field strains compared to the laboratory reference strain as shown in Figure 4. Small but distinct number of proteins showed significant expression differences between the isolates and the reference strains were selected following statistical analysis. Hierarchical clustering of these proteins (Figure 5) indicates that they can be placed into several groups. Some of these groups show consistent expression profiles across the three different field isolates as indicated by similar color in all three columns. Interestingly, the field isolates were randomly selected from separate animals, and would not be expected to be identical strains, although all share recent exposure to the host environment. We posit that in fact the similar profiles detected here are a result of the demands of the host environment, inducing change in the expression of proteins that assist survival in the host.

Sixty six proteins demonstrated a significant increase in expression in the field isolates compared to the reference strain (Table 2). Eight of these proteins were significantly increased in all three field isolates. None of the proteins with increased expression was a product of a gene unique to the field isolates. Yet, this approach allowed us to detect differences in protein expression between isolates that were not readily apparent at the genome level. Furthermore, we were able to find evidence for the presence of several protein isoforms reflecting specific SNPs that were identified by genome sequencing. For example, SNP in galactokinase gene in field isolate Cptb_RLC001_001 at position 219 generated a substitution of the original proline to a valine residue (Protein ID: Cptb_RLC_001_00052). The unique peptide containing the valine was identified by MS/MS only in this strain (Supplementary Figure 1, Fragmentation Spectra) while the original proline containing peptide of galactokinase was identified in all other strains (Cptb_C231_01684; Cptb_RLC_003_00699; Cptb_RLC_002_00731; and Uniprot: tr|D9Q9R6|D9Q9R6_CORP2 shown in Supplementary Figure 1, Fragmentation Spectra). The presence of the valine 219 in Cptb_RLC_001_00052 is also reflected in the quantitative analysis that shows that this protein is present only in isolate Cptb_RLC001 and that it is highly overexpressed relative to the reference strain. Closer examination of the results (Supplementary Table 2, ReesDiMetalldb.xlsx–row 1096) show that this is the only unique peptide identified for this protein and the remainder of the peptide repertoire for this protein are shared with the other sequences of galactokinase (Cptb_C231_01684; Cptb_RLC_003_00699; Cptb_RLC_002_00731; and Uniprot: D9Q9R6) and expressed in all strains at similar level (Supplementary Table 2, ReesDiMetalldb.xlsx–row 1204).

**Table 2:** **Proteins identified with increased abundance in the recently isolated field strains in contrast to the laboratory reference strain**.

**Protein ID**	**Gene**	**Locus**	**Protein description**	log_2_ values of increased abundance compared to Cptb_C231 (significant = ^*^)			**Predicted function by COG**
				**Cptb_RLC001**	**Cptb_RLC002**	**Cptb_RLC003**	
D9QD79	CpC231_0024	CpC231_0024	Insertion element protein	0.314		1.687^*^	Replication and repair
D9QDJ7	piuB	CpC231_0072	Uncharacterized iron-regulated membrane protein	1.509^*^		2.303^*^	Function unknown
D9QDK7	ulaA	CpC231_0082	Ascorbate-specific permease IIC component ulaA	−0.319	1.199^*^	0.835	Function unknown
D9QDP1	troA	CpC231_0116	Periplasmic zinc-binding protein troA	0.799^*^	0.648	0.614	Inorganic ion transport and metabolism
D9QDT5	deoD	CpC231_0163	Purine-nucleoside phosphorylase	1.892^*^	−0.914	0.979	Nucleotide transport and metabolism
D9QDT7	deoC	CpC231_0165	Deoxyribose-phosphate aldolase	1.487^*^	0.673	2.148^*^	Nucleotide transport and metabolism
D9QDT8	pmmB	CpC231_0166	Phosphoglucosamine mutase	1.560^*^	0.646	2.103^*^	Carbohydrate transport and metabolism
D9QE16	sdhC	CpC231_0245	Succinate dehydrogenase cytochrome b556 subunit		2.059^*^	2.821^*^	
D9QE18	sdhB	CpC231_0247	Succinate dehydrogenase iron-sulfur subunit	0.017	1.705^*^	1.702^*^	Energy production and conversion
D9QE59	ccdA	CpC231_0288	Cytochrome c-type biogenesis protein CcdA	2.695^*^			Posttranslational modification
D9QE65	ccsA	CpC231_0294	Cytochrome c biogenesis protein CcsA	0.998^*^			Posttranslational modification
D9QE78	ldh	CpC231_0307	L-lactate dehydrogenase	−0.209	1.437^*^	1.364	Energy production and conversion
D9QE89	CpC231_0319	CpC231_0319	Uncharacterized protein	0.340	1.299^*^	0.653	
D9QE90	CpC231_0320	CpC231_0320	ABC-type metal ion transport system	−0.250	1.354^*^	1.615^*^	Inorganic ion transport and metabolism
D9QE94	CpC231_0324	CpC231_0324	Uncharacterized protein	0.406	1.297^*^	−0.001	
D9QEB4	CpC231_0345	CpC231_0345	Uncharacterized protein	−0.209	2.552^*^	0.381	Function unknown
D9QED0	oppDF1	CpC231_0361	Oligopeptide transport ATP-binding protein	−0.309	1.187^*^	1.416^*^	Posttranslational modification
D9QEP1	pyc	CpC231_0477	Pyruvate carboxylase	1.120^*^	1.723^*^	1.611^*^	Energy production and conversion
D9QEQ2	rbsR	CpC231_0488	Ribose operon repressor	0.751^*^	0.892	1.014	Transcription
D9QER5	malE	CpC231_0501	Maltose/maltodextrin transport system substrate-binding protein	0.901^*^	1.401^*^	−0.334	Carbohydrate transport and metabolism
D9QEV8	uvrD	CpC231_0544	DNA helicase		1.812^*^	0.666	Replication and repair
D9Q9A8	amtR	CpC231_0651	TetR family regulatory protein	0.767^*^	0.387	1.415^*^	Transcription
D9Q9C3	CpC231_0666	CpC231_0666	5-formyltetrahydrofolate cyclo-ligase	1.354^*^	1.300^*^	1.530^*^	Coenzyme transport and metabolism
D9Q9D7	rpfB	CpC231_0680	Resuscitation-promoting factor RpfB	0.753^*^	−0.096	−1.007	Function unknown
D9Q9E8	gapA	CpC231_0692	Glyceraldehyde-3-phosphate dehydrogenase	0.843^*^	0.394	0.669	Carbohydrate transport and metabolism
D9Q9J4	glpX	CpC231_0738	Fructose-1,6-bisphosphatase	1.308^*^	1.740^*^	1.815^*^	Carbohydrate transport and metabolism
D9Q9R3	CpC231_0807	CpC231_0807	Sodium/solute symporter	2.099^*^			Posttranslational modification
D9Q9T2	CpC231_0827	CpC231_0827	Uncharacterized protein	0.334	1.982^*^	2.146^*^	Function unknown
D9Q9T3	lutB	CpC231_0828	Lactate utilization protein B	0.234	1.888^*^	2.065^*^	Energy production and conversion
D9Q9T4	lutA	CpC231_0829	Lactate utilization protein A	0.239	1.848^*^	2.476^*^	Energy production and conversion
D9Q9Y9	CpC231_0884	CpC231_0884	Uncharacterized protein	1.794^*^			
D9QAC0	cobG	CpC231_1019	Precorrin-3B synthase	0.773^*^	1.959^*^	1.946^*^	Inorganic ion transport and metabolism
D9QAC1	cobH	CpC231_1020	Precorrin-8X methyl mutase	−0.111	1.440^*^	1.324	Coenzyme transport and metabolism
D9QAC2	cobJ	CpC231_1021	Precorrin-3B C(17)-methyltransferase	−0.071	1.677^*^	1.679^*^	Coenzyme transport and metabolism
D9QAD2	pafB	CpC231_1031	Protein pafB	0.927^*^		−0.125	Transcription
D9QAE2	aspA	CpC231_1041	Aspartate ammonia-lyase	0.835^*^	1.018	1.664^*^	Amino acid transport and metabolism
D9QAG6	CpC231_1066	CpC231_1066	SPFH domain, band seven integral membrane protein	−0.075	1.213^*^	1.581^*^	Posttranslational modification
D9QAH4	acnA	CpC231_1074	Aconitate hydratase	0.665	1.274^*^	1.321	Energy production and conversion
D9QAQ7	CpC231_1157	CpC231_1157	Citrate lyase subunit beta-like protein	1.008^*^	1.593^*^	1.843^*^	Carbohydrate transport and metabolism
D9QAQ8	CpC231_1158	CpC231_1158	MaoC-like dehydratase	0.746^*^	0.737	0.789	Lipid transport and metabolism
D9QAU4	rnd	CpC231_1194	Ribonuclease D		1.427^*^		Translation ribosomal structure and biogenesis
D9QAX8	ptsF	CpC231_1229	PTS system fructose-specific EIIABC component	−0.108	2.542^*^	2.665^*^	Carbohydrate transport and metabolism
D9QBD3	ftsQ	CpC231_1388	Cell division protein FtsQ	1.030^*^	0.029		Cell wall/membrane/envelope biogenesis
D9QBF3	CpC231_1408	CpC231_1408	Transcription regulator	0.978^*^	1.194^*^	1.055	
D9QBI3	lipB	CpC231_1439	Octanoyltransferase	1.413^*^	2.008^*^	0.564	Coenzyme transport and metabolism
D9QBL7	CpC231_1476	CpC231_1476	Uncharacterized protein	0.777^*^	1.050		
D9QBM2	glnA2	CpC231_1482	Glutamine synthetase II	−0.085	1.308^*^	0.912	Amino acid transport and metabolism
D9QBQ8	CpC231_1518	CpC231_1518	HTH-type transcriptional regulator	0.451	1.193^*^	2.490^*^	Transcription
D9QC05	ycaO	CpC231_1619	Uncharacterized protein ycaO	1.034^*^	1.378^*^	2.095^*^	Function unknown
D9QC06	CpC231_1620	CpC231_1620	Nitroreductase		2.692^*^	2.402^*^	Energy production and conversion
D9QC07	CpC231_1621	CpC231_1621	Lantibiotic dehydratase	0.549	1.898^*^	2.185^*^	
D9QC08	CpC231_1622	CpC231_1622	Uncharacterized protein	0.295	1.403^*^	1.160	
D9QC16	pcsA	CpC231_1630	Carbon starvation protein A	2.049^*^	0.451	0.952	Signal transduction mechanisms
D9QC24	ykuD	CpC231_1638	L,D-transpeptidase YkuD			1.515^*^	Function unknown
D9QC80	cydA	CpC231_1695	Cytochrome d ubiquinol oxidase subunit 1	0.268		1.523^*^	Energy production and conversion
D9QCD7	CpC231_1756	CpC231_1756	Uncharacterized protein	1.445^*^	0.921	−0.298	
D9QCK3	pknG	CpC231_1823	Serine/threonine-protein kinase PknG	−0.091	0.562	1.541^*^	Signal transduction mechanisms
D9QCL1	dsbB	CpC231_1831	Disulfide bond formation protein, DsbB family		1.614^*^	1.971^*^	Posttranslational modification
D9QCN8	glpD	CpC231_1859	Glycerol-3-phosphate dehydrogenase	0.097	1.358^*^	1.393^*^	Energy production and conversion
D9QCR2	CpC231_1885	CpC231_1885	Membrane protein	1.172^*^	1.026	0.107	
D9QCY5	glpT1	CpC231_1960	Glycerol-3-phosphate transporter	0.360	1.026	1.592^*^	Carbohydrate transport and metabolism
D9QCY9	yvrC	CpC231_1964	ABC transporter substrate-binding lipoprotein yvrC	0.775^*^	2.659^*^	2.942^*^	Inorganic ion transport and metabolism
D9QD08	deoA	CpC231_1984	Thymidine phosphorylase	−0.547	1.714^*^	1.673^*^	Nucleotide transport and metabolism
D9QD98	CpC231_2034	CpC231_2034	UPF0176 protein	1.064^*^	1.016	1.411^*^	Posttranslational modification
D9QDB6	CpC231_2052	CpC231_2052	Uncharacterized protein	1.998^*^	1.661^*^	0.803	
Cptb_C231_00414	GI503006038		Hypothetical protein	1.126^*^	2.302^*^	1.222^*^	

All three proteins that were found to be more abundant in the label-free experiment (Figure 3) were also found to be highly expressed in the field isolates when using dimethylation labeling. The hypothetical protein GI: 503006038 showed higher abundance in all three isolates and pstF was highly expressed only in Cptb_RLC_002 and Cptb_RLC_003, pcsA was shown to have increased abundance (~two fold) in Cptb_RLC_001 and Cptb_RLC_003.

Several groups of proteins which have coding genes located adjacent to each other in the bacterial chromosome appeared to be more abundant in the field isolates (Table 2). Many of these were associated with metabolism. This includes three lactate utilization proteins encoded by genes CpC231_0827 to CpC231_0829, which demonstrated increased abundance in all three field isolates in contrast to the reference strain. CpC231_0829 is lactate utilization protein A (LutA), CpC231_0828 is lactate utilization protein B (LutB) while CpC231_0827 is an uncharacterized protein. BLAST and homolog search for CpC231_0827 show that this protein includes a 5-formyltetrahydrofolate cyclo-ligase domain found in enzymes involved in folate metabolism and also Lactate utilization protein C (LutC) and LutB. This indicated that this operon is probably the lutABC identified in other species (Hwang et al., 2013). This operon has been observed to be activated, and the expression of these proteins elevated, in response to altered nutrient availability in the host (Gerstmeir et al., 2003). The availability of altered carbon sources in host tissue may similarly explain the increased abundance of carbon starvation protein (pcsA).

Another cluster of proteins with increased expression in the field isolate is encoded in the gene region from CpC231_1019 to CpC231_1021. All of these have been predicted to reside in a shared operon (http://www.coryneregnet.de) and are involved with Precorrin. These genes have homologs in the cob gene products of *M. tuberculosis* (genes Rv2064 to Rv2066) and contribute to biochemical pathways involved in cobalamin and vitamin B12 synthesis (Raux et al., 2000). The PTS system fructose-specific EIIABC component (pstF) was found to be increased in abundance by both quantitative proteomic methods in our study. This system has been described to be carbohydrate regulated in the closely related *C. glutamicum*, in which it both responds to alternate carbon sources and facilitates up take of alternate carbohydrates (Ikeda, 2012).

Another group of proteins that share the same operon control and show increased abundance in the field isolates are those coded by the genes CpC231_1619 to 1622. These proteins are annotated as ycaO, the ribosomal protein S12 methylthiotransferase accessory factor (CpC231_1619), Nitroreductase (CpC231_1620) member of the Lantibiotic dehydratase family (CpC231_1621) and uncharacterized protein (CpC231_1622). BLAST and homolog searches reveal that the later contains a thiopeptide-type bacteriocin biosynthesis domain, consistent with a lantibiotic dehydratase domain. These four proteins and domains are involved in thiopeptide biosynthesis (Li et al., 2012) Indicating that this operon and the synthesis of thiopeptide are activated in the field isolate at much higher level than in the reference strain.

Fifty-six proteins were less abundant in the field isolates (Table 3) in comparison to the reference strain of which four were decreased in all three field isolates. Many of these proteins are involved in metabolic processes including two glutamate binding proteins (GluA and B), and an iron binding protein (fhuD) and a phosphate binding protein (pstS), suggesting that the field isolates have been primed to select for different nutrients. Similar to the clusters of proteins with elevated abundance, there were also proteins which are encoded by the same operon which were decreased in abundance. This includes the operon between CpC231_1833 and CpC231_1835, although the specific function of these proteins is unknown all three of them were reported to be exported proteins (Silva et al., 2013).

**Table 3:** **Proteins identified with significantly decreased abundance in the recently isolated field strains in contrast to the laboratory reference strain**.

**Protein ID**	**Gene**	**Locus**	**Protein description**	**log_2_ values of decreased abundance**			**Predicted function by COG**
				compared to Cptb_C231 (significant = ^*^)			
				**Cptb_RLC001**	**Cptb_RLC002**	**Cptb_RLC003**	
Cptb_C231_00778	Cp31_0599	Cp31_0599	hypothetical protein	−1.205^*^	−2.560^*^		
D9QDJ0	CpC231_0064	CpC231_0064	Lysozyme M1	−0.099	−0.173	−1.368^*^	Cell wall/membrane/envelope biogenesis
D9QDQ0	CpC231_0126	CpC231_0126	Glyoxalase/Dihydroxybiphenyl dioxygenase	−0.281	−0.943^*^	−1.297^*^	General function prediction only
D9QDQ6	pdxT	CpC231_0132	Pyridoxal 5′-phosphate synthase subunit PdxT	−1.452^*^	−0.796	0.654	Coenzyme transport and metabolism
D9QDR1	mmpL11	CpC231_0137	Uncharacterized protein	−2.842^*^			General function prediction only
D9QDU5	CpC231_0173	CpC231_0173	Surface antigen			−1.217^*^	
D9QDW8	CpC231_0196	CpC231_0196	Uncharacterized protein	−0.537	−0.765	−1.913^*^	
D9QDZ8	cspA	CpC231_0227	Cold-shock protein	−0.223	−0.090	−1.352^*^	Transcription
D9QE05	CpC231_0234	CpC231_0234	Secreted hydrolase	0.139	−0.448	−1.603^*^	Amino acid transport and metabolism
D9QE11	slpA	CpC231_0240	Surface layer protein A	−0.052	−0.399	−1.462^*^	General function prediction only
D9QE86	nemA	CpC231_0316	N-ethylmaleimide reductase	−0.269	−0.514	−1.300^*^	Energy production and conversion
D9QEF8	CpC231_0391	CpC231_0391	L,D-transpeptidase catalytic domain, region YkuD	0.604	−0.590	−1.639^*^	Function unknown
D9QEL9	htaA	CpC231_0454	Cell−surface hemin receptor	−0.567	−0.942^*^		
D9QEM3	htaC	CpC231_0458	Uncharacterized protein	0.030	−0.838	−1.610^*^	
D9QEQ0	maf	CpC231_0486	Maf-like protein CpC231_0486	−0.886^*^	0.324	−0.300	Cell cycle control
D9QEQ5	pccB2	CpC231_0491	Propionyl-CoA carboxylase beta chain 2	0.201	−2.348^*^	−2.676^*^	Lipid transport and metabolism
D9QEV7	CpC231_0543	CpC231_0543	Uncharacterized protein	−0.923^*^	−1.119^*^	−0.183	
D9Q935	CpC231_0574	CpC231_0574	Methylmalonyl-CoA carboxyltransferase 1.3S subunit	−1.088^*^	−0.381	−0.228	Lipid transport and metabolism
D9Q954	rpfA	CpC231_0595	Resuscitation-promoting factor	0.724	−0.020	−1.458^*^	
D9Q998	pcrA	CpC231_0641	DNA helicase	0.070	−1.359^*^	−1.895^*^	Replication recombination and repair
D9Q9A3	gluA	CpC231_0646	Glutamate ABC transporter domain-containing ATP-binding protein	−1.015^*^	−2.494^*^	−1.443^*^	Amino acid transport and metabolism
D9Q9A4	gluB	CpC231_0647	Glutamate-binding protein GluB	−0.990^*^	−2.238^*^	−1.513^*^	Amino acid transport and metabolism
D9Q9H2	gppA2	CpC231_0716	Ppx/GppA phosphatase family	−1.726^*^	−2.633^*^	−0.665	Inorganic ion transport and metabolism
D9Q9L9	sprX	CpC231_0763	Trypsin-like serine protease	0.088	−0.902	−2.282^*^	Posttranslational modification
D9Q9R9	CpC231_0813	CpC231_0813	Glyoxalase/Dihydroxybiphenyl dioxygenase	−0.710	−1.072^*^	−0.552	Function unknown
D9Q9T0	CpC231_0824	CpC231_0824	Uncharacterized protein	−0.893^*^	−1.360^*^	−0.543	
D9Q9Y3	fhuD	CpC231_0878	Iron(3+)-hydroxamate-binding protein fhuD	−1.756^*^	−1.569^*^	−1.711^*^	Inorganic ion transport and metabolism
D9QA16	gpsA	CpC231_0913	Glycerol-3-phosphate dehydrogenase	0.005	−1.388^*^	0.405	Energy production and conversion
D9QA35	ptsG	CpC231_0932	Phosphotransferase system II Component	−0.800^*^	−0.370	0.481	Carbohydrate transport and metabolism
D9QAE1	fhs	CpC231_1040	Formate–tetrahydrofolate ligase	−1.007^*^	0.355	0.293	Nucleotide transport and metabolism
D9QAL4	ribD	CpC231_1114	Riboflavin biosynthesis protein ribD	−1.737^*^			Coenzyme transport and metabolism
D9QAL9	priA	CpC231_1119	Primosomal protein N	−0.786^*^			Replication recombination and repair
D9QAN5	efp	CpC231_1135	Elongation factor P	−0.110	−1.051^*^	−0.304	Translation ribosomal structure and biogenesis
D9QB52	pyrH	CpC231_1306	Uridylate kinase	−0.879^*^	0.476	0.459	Nucleotide transport and metabolism
D9QBH1	CpC231_1426	CpC231_1426	Iron-sulfur cluster insertion protein erpA	−0.371	−2.254^*^	−0.648	Function unknown
D9QBL4	CpC231_1473	CpC231_1473	Uncharacterized protein	−0.052	−0.227	−1.565^*^	Function unknown
D9QBM5	CpC231_1485	CpC231_1485	Uncharacterized protein	0.115	−0.299	−1.459^*^	
D9QBU6	CpC231_1558	CpC231_1558	Uncharacterized protein	−0.325	−1.561^*^	−1.290^*^	General function prediction only
D9QBZ1	CpC231_1605	CpC231_1605	Uncharacterized protein	0.294	−0.116	−1.637^*^	
D9QC41	CpC231_1655	CpC231_1655	Uncharacterized protein	−0.163	−1.052^*^	0.280	
D9QC90	pstS	CpC231_1706	Phosphate-binding protein PstS	−0.789^*^	−2.400^*^	−2.551^*^	Inorganic ion transport and metabolism
D9QCE7	iunH2	CpC231_1766	Inosine-uridine preferring nucleoside hydrolase	−1.783^*^	−0.735	−1.189	Nucleotide transport and metabolism
D9QCH2	lipY	CpC231_1792	Secretory lipase	−0.629	−0.755	−1.690^*^	General function prediction only
D9QCJ4	def	CpC231_1814	Peptide deformylase	−0.287	−1.257^*^		Translation ribosomal structure and biogenesis
D9QCL3	CpC231_1833	CpC231_1833	Uncharacterized protein	−0.788^*^	−0.725	−0.672	
D9QCL4	CpC231_1834	CpC231_1834	Uncharacterized protein	−1.593^*^	0.453		
D9QCL5	CpC231_1835	CpC231_1835	Uncharacterized protein	−1.309^*^	−0.473	−0.251	
D9QCM3	CpC231_1843	CpC231_1843	Uncharacterized protein	−1.305^*^			
D9QCM5	CpC231_1845	CpC231_1845	Uncharacterized protein	0.905^*^	−0.335	−1.832^*^	
D9QCN2	CpC231_1852	CpC231_1852	Sdr family related adhesin	−1.734^*^			
D9QCR4	hspR	CpC231_1887	Heat shock protein HspR	−0.867^*^			Transcription
D9QCX0	ppsA	CpC231_1945	Phthiocerol synthesis polyketide synthase type I PpsA	−0.073	−1.249^*^	−1.193	Lipid transport and metabolism
D9QCX4	cmtC	CpC231_1949	Trehalose corynomycolyl transferase C	−0.139	−0.382	−1.470^*^	General function prediction only
D9QCY4	glpQ	CpC231_1959	Glycerophosphoryl diester phosphodiesterase	−0.412	−2.820^*^	−2.898^*^	Energy production and conversion
D9QD39	CpC231_2016	CpC231_2016	Cation transport protein	−0.331	−0.089	−1.344^*^	Inorganic ion transport and metabolism
D9QDE3	trpC	CpC231_2079	Bifunctional indole-3-glycerol phosphate synthase	−0.114	−1.241^*^	−0.166	

*C. pseudotuberculosis* is an animal pathogen that has not been extensively studied however many of the proteins we identified as differentially abundant in field strains had homologs in *M. tuberculosis*, a significant human pathogen. These include proteins that are known to be differentially regulated in a hypoxic environment on the basis of transcriptional profiling, such as the increased expression of Nudix hydrolase (nudL; Park et al., 2003), pyruvate carboxylase, fructose-1,6-bisphosphatase (glpX; Rustad et al., 2009) and decreased expression of riboflavin biosynthesis protein RibD (Rustad et al., 2008). There is also a pair of more abundant proteins that are encoded by adjacent genes CpC231_0165 and CpC231_0166 which have homologs deoC and pmmB in *M. tuberculosis*. The protein product of pmmB has been suggested as contributing to the production of mannose in the mycobacterial cell wall (Mishra et al., 2012), a significant bacterial defense against the host.

A Clusters of Orthologous Groups analysis was performed on these proteins to predict likely function. A functional group could be predicted for 94 of the proteins that had demonstrated significant alterations of abundance in the field isolates. The remaining 30 did not have any predicted function. The majority of these proteins had some sort of metabolic function including utilization of metabolites such as amino acids and lipids (Tables 2, 3). Energy production and conservation was the functional group with the greatest number of proteins allocated, suggesting that the field isolates had needed to alter their metabolism in response to the recent host environment.

## Discussion

This aim of this work was to characterize the proteomic differences between *C. pseudotuberculosis* isolated from naturally infected sheep and a laboratory reference strain. While infection of cultured cell lines is commonly utilized to study bacterial genomic and proteomic changes occurring in the host cell (Shi et al., 2006; Pávková et al., 2013) there are fewer studies of bacteria isolated from infected host (Twine et al., 2006; Schmidt and Volker, 2011; Rees et al., 2015). One of the challenges in such studies is to collect sufficient amount of bacteria to allow thorough proteomics analysis (Schmidt and Volker, 2011).

In this study we used a different approach to address this technical challenge, simply by culturing freshly isolated bacteria in BHI media and compare it to a laboratory reference strain of *C. pseudotuberculosis* growing under identical conditions. The adaptation of the isolated field strains to their source growth conditions persisted despite brief culturing in liquid media and this allowed us to detect clear and distinguishable changes between the field strains and the laboratory reference strain.

Mass spectrometry based proteomic analyses are currently limited to identification of several thousands of proteins (Richards et al., 2015). This might be a limitation for studies of eukaryotes and mammals but should not affect studies of prokaryotic organisms with relatively small genomes such as *C. pseudotuberculosis*. Indeed, our results have demonstrated that almost complete proteome coverage can be achieved. We identified and quantified >1350 proteins and >60% of the predicted ORFs which is providing significantly wider coverage of *C. pseudotuberculosis* genome compared to previous reports (Silva et al., 2014).

In this study the genomic and the proteomic mappings provide similar general conclusions regarding the similarity between the field strains and reference strain. We note that >10% of genes differed between sequences due to the presence of SNPs or the presence or absence of genes. Similarly, about 10% of the protein repertoire differed in abundance between the samples. However, the proteins that differed in abundance were not those predicted to be transcribed by altered genes but were transcribed of genes shared and conserved across all isolates and reference strain. The field isolates were collected from the same geographical area so it is possible that these strains share some genetic changes or epigenetic mechanism that does not present in the reference strain that might lead to the observed proteomic differences. However, the relevant geographic area is rather large (South western Victoria consist of ~60,000 km^2^) and the field isolates were collected from different animals on different time points, which reduces the feasibility of these options as well as our genetic data indicating very high similarity in between the field isolates and reference strains (Figure 2).

The isolated bacteria were cultured for a relative short time in synthetic media prior to genetic and proteomic analyses. It is possible, that under these less-stressed conditions, not all of the proteomic changes that occur in host will appear. Yet, the “stress-relief”-related changes that can be detected during the adaptation of the field isolates to the synthetic media are useful indicators to the mechanisms the bacteria are using in order to survive and thrive within the host. As the field strains were recently isolated from sheep lymph nodes it can be postulated that proteins involved in resistance to the hostile environment of the caseous lymph node may be primed to have an increase in expression. *C. pseudotuberculosis* residing in caseous lymph nodes will often be consumed by macrophages, but survive this phagocytosis and reside in the lysozyme compartment which is known to be hypoxic (Pacheco et al., 2012) Protein repertoires have been demonstrated to differ between hypoxic and normally growing *M. tuberculosis* (Wolfe et al., 2013). Therefore, it is not surprising that proteins that are implicated in dealing with a hypoxic environment such as pyc and glpX (listed in Table 2) are present in increased quantities in strains recently isolated from lymph nodes, compared to strains which have been passaged many times in liquid media that has been aerated by continuous shaking.

Bacterial liquid culture media, such as BHI, have readily available carbon sources such as carbohydrates however bacterial growth within the host lymph node may require the utilization of alternate nutrients. This could be done by several proteins derived by the sdhABC operon encoding succinate dehydrogenase (CpC231_0245 to CpC231_0247). It is of interest that succinate dehydrogenase proteins were found to be elevated in the field isolates, as these enzymes have previously been noted to be a significant aspect of the metabolic pathways evoked when bacteria have to adapt to different environments. Specifically, SDH expression is seen to be increased in response to hypoxic conditions (Inui et al., 2004) and during growth on metabolic substrates other than glucose such as lactate and acetate (Gerstmeir et al., 2003; Bussmann et al., 2009). SDH expression is also part of the enzymatic switch from glucose to acetate (Wolfe, 2005). The mechanism by which this occurs is not fully elucidated but was reported to be controlled by several regulatory systems, including that expression induction by RamA and repression by GlxR (Bussmann et al., 2009). Elevated abundance of proteins such as succinate dehydrogenase suggests that these field isolates have adapted to the environment of the host and more readily produce these proteins than laboratory strains. In our analysis no DNA sequence changes were noted in either GlxR (CptbC231_0208) or CpC231_0244 which is a transcriptional regulator that bears homology to RamB. Interestingly, there was one SNP in the coding region of the serine proteases family genes at CpC231_0239 which is considered a response regulator by bioinformatics prediction (Caspi et al., 2014). Further qPCR studies of the genes encoding the differentially expressed proteins would be needed to determine the mechanism.

An intriguing discovery is the increased expression of proteins from the gene cluster encoding thiopeptide biosynthesis (genes CpC231_1619 to 1622–Table 2). These uniquely modified peptides have antibacterial activity as well as anti-tumor activity and other capabilities. The antibacterial activity of thiopeptides suggests these peptides may be used to obtain advantage over other bacterial species and dominate their ecological niche (Ruhe et al., 2013). The increased abundance of thiopepetide synthesis cluster proteins seen in the field isolates may contribute to their dominance in the host. The extent to which competition between bacterial commensals occurs in lymph nodes is unclear. In contrast to mammalian host surfaces such as gut and upper airways, which normally host diverse populations of bacteria, any bacteria transported to the lymph nodes via the lymphatic circulation are quickly attacked by the abundant resident immune cells particularly phagocytes (von Andrian and Mempel, 2003). However, certain pathogens can replicate at this site in disease. There is evidence at the transcriptional level of microbes in apparently healthy lymph nodes which supports their role as concentrators of the commensal, endemic, and potential pathogenic microbial communities of a host species (Wittekindt et al., 2010). Therefore, the capacity of a microorganism to generate an antimicrobial peptide to exert its dominance over a mixed population of bacteria would certainly be an advantage for bacteria such as *C. pseudotuberculosis* when establishing infection in the inoculating wound (Dorella et al., 2006) and subsequently in the respiratory tract (Bush et al., 2012) and possibly also in the lymph nodes.

It was recently shown that six genes that are involved in the synthesis and secretion of the thiopeptide bacteriocin could modulate the immune response of dendritic cells toward the probiotic bacteria *Lactobacillus plantarum* WCFS1 (Kindrachuk et al., 2013). Furthermore, a different study revealed that nicin Z, one of the best studied thiopeptide, can induce the secretion of different chemokines and modulate the host immune response to bacterial infection (Meijerink et al., 2010). Therefore, it is possible that the increased expression of thiopeptide synthesis proteins is a mechanism that allows the bacteria to modulate the host innate immune responses. This in turn may provide these field isolates selective advantage within the host particularly within immune-reactive lymph nodes.

Decreased abundance in the field isolates compared to the laboratory reference strain was also documented (Table 3). Proteins that were shown to have significantly lower expression in all filed isolates include the phosphate-binding protein pstS also known as Phosphate ABC transporter. In *M. tuberculosis* inactivation of similar genes was linked to reduced virulence (Peirs et al., 2005) and pstC was shown to act also as adhesion molecule that can bind macrophages and promote phagocytosis (Sanchez et al., 2009). In *Corynebacterium glutamicum* this gene transcription is controlled by the RamB via phosphate sensitive regulation that involve GlxR and phoRS (Sorger-Herrmann et al., 2015) Phosphate dependency was also documented in *Clostridium acetobutylicum* where pstC expression is induced only under conditions of organic phosphate limitation (Fischer et al., 2006). It is likely that the reduced abundance of PstC we observed in the field isolates is the results of the relatively high phosphate concentration in the BHI growth media used in our experiments. Similarly, we observed decreased abundance in the field isolates of several proteins involved in metabolic processes such as glutamate utilization, iron uptake which may reflect altered availability of these substrates in the host compared with the culture media. The riboflavin biosynthesis proteins RibD was also less abundant, and this is in agreement with microarray studies in which the *M. tuberculosis* homolog ribG has been noted to be down regulated in hypoxia (Rustad et al., 2008).

A successful pathogen will need a dynamic repertoire of expressed proteins to counter the hostile environment of the host. This comprehensive quantitative whole proteome analysis comparing different isolates has identified groups of proteins that are likely to work together in response to the host environment, thus providing different and complementary information to that derived from genome sequencing. Identified proteins with increased abundance were consistent with those that could be anticipated to occur in response to the hostile host environment. These included proteins that respond to the hypoxic environment of the host as well as those involved in metabolism of a different set of carbon sources and thiopeptide biosynthesis. Yet, the differential abundance of some sets of proteins in field isolates from infected hosts is currently unexplained, further investigation of these proteins may provide information to begin to unravel the complex interaction between pathogen and host.

### Conflict of interest statement

The authors declare that the research was conducted in the absence of any commercial or financial relationships that could be construed as a potential conflict of interest.

## Supplementary material

Supplementary Table 1 **ReesLFQ.xlsx**.

Supplementary Table 2 **ReesDiMet.xlsx**.

Supplementary Table 3 **Fasta files whole sequence predicted proteomes of all isolates**.

Supplementary Figure 1 **MS/MS spectra of galactokinase SNP**.
