# Supplementary figures and images for: Changes in protein abundance are observed in bacterial isolates from a natural host
Source: Front Cell Infect Microbiol. 2015 Oct 14;5:71. doi: 10.3389/fcimb.2015.00071 (PMC4604328; doi:10.3389/fcimb.2015.00071)

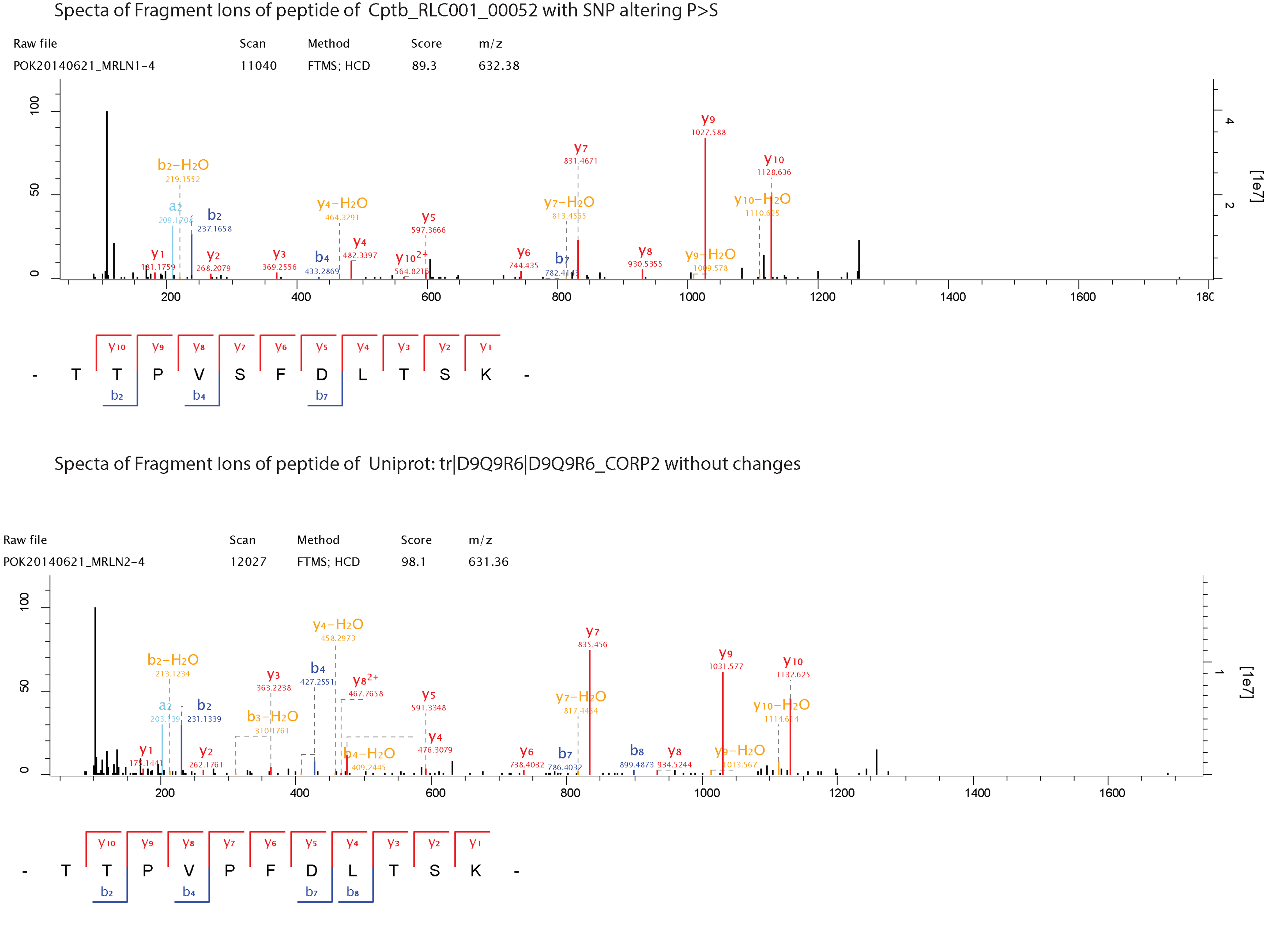

Supplement: Supplementary Figure 1 — MS/MS spectra of galactokinase SNP. [file Image1.TIF]
